# Current insights into the aetiology of adolescent idiopathic scoliosis

**DOI:** 10.1007/s00402-017-2756-1

**Published:** 2017-07-14

**Authors:** Michal Latalski, A. Danielewicz-Bromberek, M. Fatyga, M. Latalska, M. Kröber, P. Zwolak

**Affiliations:** 1 Department of Paediatric Orthopaedics, Medical University of Lublin, ul. Gebali 6, 20-093 Lublin, Poland; 2 Department of Vitreoretinal Surgery, Medical University of Lublin, ul. Chmielna 1, 20-079 Lublin, Poland; 3 Department of Orthopaedics, Trauma und Spine Surgery, Asklepios Klinik Altona, Paul-Ehrlich-Strasse 1, 22763 Hamburg, Germany

**Keywords:** Scoliosis, Scoliosis aetiology, Spine deformity, Spine multifactorial growth

## Abstract

Scoliosis occurs in about 0.2–0.6% of the general population. In the majority of cases the cause of this entity remains mostly unidentified. The search for the causes covers almost all aspects of its possible origin. We collected and systematised the contemporary theories and concepts concerning the aetiology of adolescent idiopathic scoliosis. Genetic and hereditary factors are commonly accepted as possible causes; however, the identification of the single gene responsible for the development of this condition seems impossible, which suggests multifactorial mechanism of its formation. Dysfunctions of the nervous system are recognised risks related to the development of scoliosis, but they are classified as belonging to a separate aetiological category. Scoliosis develops at the quickest rate during the child’s growth spurt, which prompted the research on the role of the growth hormone in scoliosis aetiology. Melatonin is another hormone that is studied as a possible factor involved in development of this entity. In cases of progressive scoliosis, increased activity of calmodulin—a protein that regulates the levels of calcium ions—has been observed. The scientists have characterised numerous qualitative and quantitative changes in the composition of the tissue of intervertebral discs, spinal ligaments and paraspinal muscles. Some of the theories, explaining the nature of this entity, presented in this review seem to have only a purely theoretical value; their proliferation only confirms the fact that the actual nature of this condition has not been unveiled yet, and suggests its multifactorial aetiology.

## Introduction

Scoliosis is found in about 0.2–0.6% of the general population, and in the majority of cases its cause remains unidentified; therefore, 70–90% of the cases of scoliosis are classified as idiopathic [1]. Its dominant feature is the fact that it develops spontaneously during the growth of the child, showing a progressive tendency. The question about the reasons of adolescent idiopathic scoliosis (AIS) has been puzzling the researchers for over 50 years now; yet there is still no clear answer in sight. The search for the causes covers almost all aspects of its possible origin—genetic, environmental, hormonal, metabolic, biochemical, neurological, and asymmetric growth. The authors of this work present an overview of theories attempting to explain the aetiopathogenesis of the AIS.

## Genetic factors

The fact that genetic factors contribute to the development of scoliosis has been widely accepted [2, 3]. Studies indicated that 11% of first-degree relatives are affected, as are 2.4 and 1.4% of second- and third-degree relatives, respectively [4]. Similar tendencies have been observed in monozygotic twins [5, 6]. The reports regarding the exact way by which the condition is passed on are still ambiguous. Justice et al. suggested the autosomal dominant mode of inheritance [7]. The study of Xiao-Yang et al. [8] demonstrates the abnormal expression of lncRNAs and mRNAs in AIS, and the expression of some lncRNAs was related to clinical features. The differential expression of lncRNAs is potentially valuable in the development of specific PCR markers and in providing more support on treatment and prognosis. However, this study has its limitations. First, although the different expression patterns of identified lncRNA genes suggest potential function in AIS pathogenesis, direct supporting evidence is lacking. Second, only four pairs of samples were used in microarray analysis. This may cause a loss of some important information and decrease the accuracy of biomarker selection. Third, RNA expression was tested in peripheral blood in this study [8].

Nowak et al. paid attention on transcriptional activity of TGF-β2, TGF-β3, and TGFBR2 and the expression profile of TGF-β-responsive genes. He noted that they differ in paravertebral muscle transcriptomes depending on the age of scoliosis onset and the side of the scoliotic curve. He suggested this phenomenon could signify a different involvement of TGF-β signalling in the pathogenesis of juvenile and adolescent curves. Analysis of TGF-β-responsive genes that differ in the concave and convex paravertebral muscle transcriptomes of AIS patients highlights the upregulation of genes localised in the extracellular region of the concave side of the curve (LTBP3, LTBP4, ITGB4, and ITGB5) [9]. This finding may suggest that the extracellular region of paravertebral muscles is an interesting target for future molecular research on AIS pathogenesis. Czeizel et al., on the other hand, proposed a multifactorial or polygenic mode of inheritance to explain the wide variability in the presentation of scoliosis amongst family members [10]. Progress in the mapping of the human genome and current genetic methodology now allows the screening of the entire genome of an individual. The authors cited many different chromosomal loci related to scoliosis, which have been identified in different families [11]. As a tool for the investigation of complex disease, genome-wide association studies (GWAS) were recently applied to the genetic research of AIS [12]. In 2013, Kou et al. identified G protein-coupled receptor 126 (*GPR126*), which can be implicated in the development of AIS [13]. The functional consequence of GPR126 was also confirmed by in vivo experiments. It was found that GPR126 knockdown zebrafish had shorter body lengths and delayed ossification of the vertebrae, as well as slower escape responses, indicating possible neurological defects [13].

Recently, Sharma et al. [12] performed transmission-disequilibrium tests in 419 AIS families, which identified associated single nucleotide polymorphisms (SNPs) in the proximity of the cell adhesion molecule with homology to the L1CAM (CHL1) gene. They genotyped additional SNPs in chromosome 3p26.3 and tested the association in two follow-up case–control cohorts. They obtained the strongest association in rs10510181 with all three cohorts combined. *CHL1* encodes an axon guidance protein related to Robo3, mutation of which could lead to horizontal gaze palsy with progressive scoliosis (HGPPS), a rare disease marked by severe scoliosis [14].

Despite continuous efforts, so far no single gene exclusively related to scoliosis has been found [15]. A global meta-analysis of genome-wide association study (GWAS) data should be helpful for the elucidation of genetic loci related to AIS. It seems that scoliosis is a complex genetic disorder, with one or more genes involved, which together with environmental factors can lead to spinal deformities.

There are, however, some tests which can estimate the probability of the progression of the deformity [16]. The risk of the progression score can be calculated using these saliva-based DNA markers that stratify risk for the patients on a scale of 1–200. The test cannot answer the question regarding the cause of the condition, but it allows the making of personalised medical decisions for treatment algorithms, and improves the quality of care.

## Hormonal factors

### Growth hormone

It is well recognised that scoliosis progresses at the highest rate during the adolescent growth spurt. The observation that the growth hormone plays a role in the progression of scoliosis also appears to be rather obvious. Increased levels of the growth hormone have been found in adolescent girls [17]. Additionally, some cases of a significant progress of scoliotic curvature have been reported in patients undergoing growth hormone therapy [18]. However, this observation was not confirmed by Misol et al., who found no differences in the growth hormone levels between patients with scoliosis and the clinical control group [19].

### Estrogens

A higher progression rate in adolescent females than males suggests that sex hormones may have some influence on AIS progression curve. It indicates that estrogens have a role in its onset and development. This role of oestrogen seems possible because of its interaction with factors that influence the development and progression of this spinal deformity. Despite the fact that estrogens are not causative factors of AIS, they could impact the progression of spinal deformity by interacting with factors that modulate bone growth, biomechanics and structure [20].

### Melatonin

Recently, melatonin, a hormone secreted mainly by the pineal gland, has caused a great interest among scoliosis aetiology researchers [21]. Machida et al. concluded that spinal deformation in chickens induced by the removal of pineal gland produces a condition similar to idiopathic scoliosis, with the vertebral rotation and rib humps found in humans [22]. They also claimed that the development of scoliosis could be prevented by an intramuscular implantation of the pineal gland or an intraperitoneal injection of melatonin [22, 23]. Additionally, bipedal rats that underwent pinealectomy, which walk only on their hind limbs in an upright posture, which can be achieved by removal of their forelimbs, developed scoliosis, while normal quadrupedal rats did not [23]. It means that posture and gravity play an important role in the aetiopathogenesis of scoliosis. Subsequently, same researchers found, in a small study involving 30 adolescents with AIS, that patients with progressive scoliosis had a 35% decrease in melatonin levels throughout the night compared to the control group [24]. Such findings may lead to the conclusion that human idiopathic scoliosis may be due to an inherited disorder of neuro-transmitters of neuro-hormonal origin, associated with bipedal condition, where a horizontal localised neuro-muscular imbalance starts and produces the scoliotic deformity of the fibro-elastic and bony structures of the axial spinal pilar [25].

However, these results have not been replicated in other studies [19, 26, 27]. Cheung et al. compared the results of melatonin secretion suppression in chickens, achieved by means of constant exposure to light and pinealectomy [28]. Even though constant light can effectively suppress melatonin secretion in a similar manner to surgical pinealectomy, no scoliosis developed in those chickens. Therefore, a connection between the surgical procedure itself and the development of scoliosis exists. Additionally, in a pinealectomy model using primates, scoliosis could not be produced despite the suppression of melatonin in a 28-month period of observation [29]. The author suggested that the possible aetiological factors causing scoliosis in lower animals are different from primates, and findings in lower animals cannot necessarily be extrapolated to humans. Measurements of melatonin levels in patients with scoliosis have been equally controversial. Melatonin levels are known to diminish in sleep disorders, but an increased association with scoliosis has not been proven [30]. At the same time, patients with AIS do not show any sleep disorders [31].

### Calmodulin

Calmodulin is a calcium-binding receptor protein that regulates the contractile properties of muscles and platelets. According to Kindsfater et al. increased calmodulin levels in platelets have been shown to be associated with curve progression [32]. Cohen et al. [33] found a 2.5- to 3-fold increase in the activity of calmodulin in the platelets of patients with scoliosis. They suggested that the platelet calmodulin level could be a better indicator for progression of the curvature than the Risser sign alone. However, the role of calmodulin in the aetiopathogenesis of scoliosis has not been defined yet, and work in this area is still in progress.

Lowe et al. showed that platelet calmodulin levels correlate closely with curve progression and stabilisation by bracing or spine fusion. Correlation with nonprogressive curves was not as consistent, with 27% noncorrelation. [34].

To explain the relationship of platelet calmodulin levels to scoliosis curve changes in AIS brought by spontaneous correction, or by brace treatment, Lowe et al. [35] attributes the platelet calmodulin changes to paraspinous muscle activity. He suggests that the calmodulin acts as a systemic mediator on tissues having a contractile system (actin and myosin). His theory includes some controversies. First, the lack of normal data and the large variability in baseline levels of platelet calmodulin, necessitating the use of the AIS subjects as their own controls. Second, calmodulin is not usually used as a marker of platelet activation, and third, whether the platelet calmodulin changes which appear to reflect an abnormality of a portion of the spine are related to local and/or regional changes in muscles, nervous system, or immature vertebrae [35].

## Neurological abnormalities

Some authors have shown abnormalities of visual, vestibular, proprioceptive and postural control involving corpus callosum, cerebral hemispheres and the brain stem [36–38]. Lowe et al. [39] suggested that the pathogenesis of AIS results from a primary pathology in the hind brain causing a defect of central control, or processing in the central nervous system (CNS) that affects a normal growing spine. Neurological abnormalities with AIS have been explained by four fairly comprehensive concepts for pathogenesis: (1) visuo-spatial perceptual impairment producing a motor control problem [40], (2) body-spatial orientation concept [41], (3) neurodevelopmental concept [42] and (4) sensory integration disorder [43].

Burwell et al. states that AIS in girls results from developmental disharmony expressed in spine and trunk between autonomic and somatic nervous systems—double neuro-osseous theory. The autonomic component of this pathogenesis involves selectively increased sensitivity of the hypothalamus to circulating leptin (genetically determined upregulation possibly involving inhibitory or sensitising intracellular molecules, such as SOC3, PTP-1B and SH2B1, respectively), with asymmetry as an adverse response. This asymmetry is directed bilaterally via the sympathetic nervous system to the growing axial skeleton where it may initiate the scoliosis deformity (leptin–hypothalamic–sympathetic nervous system concept = LHS concept) [44].

Liang et al. suggested that leptin gene variations are not associated with AIS and low serum leptin probably is a secondary outcome which may be related to the low capability of adipogenesis in AIS. The decreased leptin receptor levels may lead to the hyposensitivity to leptin. In his opinion these findings implied that abnormal peripheral leptin signalling plays an important role in the pathological mechanism of AIS [45].

## Collagen and elastic fibres

Collagen and elastic fibres are the principal elements in the supporting structures of the spinal column, and for this reason have been the focus of many studies dealing with the pathophysiology of scoliosis. Hadley-Miller et al. noted elastic fibre abnormalities in the spinal ligaments in a considerable number of patients with AIS, compared with those of healthy individuals [46]. Studies focusing on the quality and quantity of the proteoglycan and collagen contents of the intervertebral disc have produced contradictory results. Pedrini et al. [47] demonstrated an abnormal proportion of glycosaminoglycans and collagen content of the nucleus pulposus in intervertebral discs in patients with scoliosis. This estimate was not supported by Oegema et al. [48]. Similar studies were carried out by Roberts et al. [49]. Differences in the distribution of collagen in patients with scoliosis, compared with controls, were observed. Similar studies were performed in relation to finding the changes in the paraspinal muscles. Spencer and Eccles [50] described the two types of muscle fibres in paraspinal muscles in patients with IS. Ford et al. [51] observed a substantial decrease in muscle spindles in all paraspinal muscles that were tested in patients with scoliosis.

Nowak et al. also observed alternative splicing of vitamin D receptor gene (VDR) mRNA which occurs in paravertebral muscles and blood tissue of idiopathic scoliosis patients regardless the age of onset. Authors suggest that the number of mRNA copies of VDRl isoform in paravertebral muscles of the curve concavity might be one of the factors differentiating juvenile and adolescent type of idiopathic scoliosis [52]. Although changes have been identified within the extracellular matrix, spinal ligaments and muscles, and the intervertebral disc, there is no way to determine whether these are primary factors, causing scoliosis, or secondary, resulting from the spinal deformity itself. Currently, researchers are inclined towards the latter concept [53].

## Biochemical factors

Biomechanical factors have been recognised to play a significant role in the progression of spinal deformities. According to Karski, scoliosis is caused by an asymmetry of the abduction of the hips with a co-occurring abduction contracture of the right hip in patients with “the Syndrome of Contractures” [54, 55]. Asymmetry in the movement of the hips during walking and standing leads to asymmetry of load and bilateral asymmetry of the development of the pelvis, subsequently resulting in a gradual development of scoliosis [31, 56].

In turn, Roaf et al. [4] proposed “a vicious cycle” theory, with asymmetrical loading from the scoliosis, producing increased compression on the concave side of the curve, which eventually decelerates growth. The reduced loading on the convex side, which accelerates growth, creates a larger deformity that accentuates the asymmetric loading and perpetuates a progression cycle. The above mechanism is based on the Hueter–Volkmann law [57]. It was tested in some studies performed by Stokes et al. [58]. According to this theory, the developed deformity continues progressing due to the increasing mechanical load and the asymmetric growth of the vertebral body. However, the linear relationship between tension and growth postulated in the Hueter–Volkmann law has been widely debated and deemed an oversimplification, as it does not explain all the changes which occur during the growth [59–61].

It appears to be evident that there are loads of various intensities and directions, impacting on the growth plate during skeletal maturation. In addition, the shapes of the cartilage itself also vary. It is, therefore, unquestionable that the development and growth of the bones is a process that takes place in very complex geometric system. Thus, the accelerated growth of adolescents, when spinal growth is rapid, is a period of time characterised by the greatest susceptibility to the progression of scoliosis [62]. The spinal mechanics, of course, cannot be treated as an aetiological factor in isolation; however, in conjunction with other factors, it does seem to contribute to the progress of spinal deformation.

## Growth imbalance

It is commonly accepted that spinal growth disturbance can induce scoliosis and cause progression of the disease, particularly in adolescence. Somerville et al. [63] introduced the concept that the development of scoliosis is related to changes in the sagittal profile. On the other hand, Smith et al. [64] described a transverse plane deformity and a bone-drift phenomenon towards the concavity of the curve. Drobyshevskiy et al. described a local internal lateral fixation (LILF) of the dura mater spinalis to the wall of the vertebral channel as the main reason of the serious idiopathic scoliosis. He found the trace of flat tension of the dura mater in conjunction between the dura matter and the periosteum of spinal canal of concave side. In addition, he found that patients with starting progression of scoliosis have LILF of the dura mater in several cases. In his opinion the magnetic resonance imaging (MRI) for a LILF of the dura mater can help to make correct decision regarding future progression of scoliosis; in addition, allowing the early treatment of scoliosis by separation of the LILF of the dura mater by overcorrection brace [65].

The sagittal plane of AIS is known to be associated with hypokyphosis. A relatively small imbalance of growth of anterior and posterior structures has been postulated as the cause [66]. According to this hypothesis, the anterior structures grow more rapidly than the posterior ones, and with bending forward, the vertebral bodies at the apex tend to move out of the way by rotating to the side.

Hitier et al. observed lateral semicircular canal asymmetry in idiopathic scoliosis. In his opinion, adolescents with idiopathic scoliosis exhibit morphological vestibular asymmetry, probably determined well before the birth. Since the vestibular system influences the vestibulospinal pathway, the hypothalamus, and the cerebellum, he suggests that this indicates that the vestibular system is a possible cause of later morphological, hormonal and neurosensory anomalies observed in AIS [67].

## Conclusions

Some of the theories described in this review seem to have only a purely theoretical value; their multitude only confirms the fact that the actual nature of this entity has not been unveiled yet, and suggests its multifactorial aetiology. Without a doubt, a single factor responsible for idiopathic scoliosis has not been identified. Moreover, there is also no way to determine whether the aforesaid changes are of a primary or secondary character. A statement that scoliosis is a multifactorial condition with genetic predisposing factors is apparently the closest to the truth.

## Abbreviations

AIS: Adolescent idiopathic scoliosis
CNS: Central nervous system
LILF: Local internal lateral fixation
MRI: Magnetic resonance imaging
VDR: Vitamin D receptor gene
